# Zinc Supplementation Alters Plasma Aluminum and Selenium Status of Patients Undergoing Dialysis: A Pilot Study

**DOI:** 10.3390/nu5041456

**Published:** 2013-04-22

**Authors:** Chih-Hung Guo, Pei-Chung Chen, Guoo-Shyng W. Hsu, Chia-Liang Wang

**Affiliations:** 1 Micro-Nutrition & Biomedical Nutrition Labs, Institute of Biomedical Nutrition, Hung Kuang University, Taichung 433, Taiwan; E-Mails: eillyguo@sunrise.hk.edu.tw (C.-H.G.); chenpc@sunrise.hk.edu.tw (P.-C.C.); 2 Department of Nutritional Science, Fu Jen University, Taipei 242, Taiwan; E-Mail: 002613@mail.fju.edu.tw; 3 Department of Nephrology, Kuang-Tien General Hospital, Taichung 433, Taiwan

**Keywords:** zinc, aluminum, selenium, oxidative stress, long-term dialysis

## Abstract

End stage renal disease patients undergoing long-term dialysis are at risk for abnormal concentrations of certain essential and non-essential trace metals and high oxidative stress. We evaluated the effects of zinc (Zn) supplementation on plasma aluminum (Al) and selenium (Se) concentrations and oxidative stress in chronic dialysis patients. Zn-deficient patients receiving continuous ambulatory peritoneal dialysis or hemodialysis were divided into two groups according to plasma Al concentrations (HA group, Al > 50 μg/L; and MA group, Al > 30 to ≤ 50 μg/L). All patients received daily oral Zn supplements for two months. Age- and gender-matched healthy individuals did not receive Zn supplement. Clinical variables were assessed before, at one month, and after the supplementation period. Compared with healthy subjects, patients had significantly lower baseline plasma Se concentrations and higher oxidative stress status. After two-month Zn treatment, these patients had higher plasma Zn and Se concentrations, reduced plasma Al concentrations and oxidative stress. Furthermore, increased plasma Zn concentrations were related to the concentrations of Al, Se, oxidative product malondialdehyde (MDA), and antioxidant enzyme superoxide dismutase activities. In conclusion, Zn supplementation ameliorates abnormally high plasma Al concentrations and oxidative stress and improves Se status in long-term dialysis patients.

## 1. Introduction

Patients with end stage renal diseases (ESRD) that require long-term dialysis are a public health concern worldwide. Despite dialysis treatment, these patients still have high morbidity and mortality rates [1]. The major contributing risk factors include wasting, an oxidant-antioxidant imbalance, progressive inflammation, impaired immune responsiveness, and infection [2]. Recent studies have shown that deficiencies in the essential trace elements zinc (Zn) and selenium (Se) are common findings, which predispose patients to the complications noted above [3,4,5,6]. However, the mechanisms underlying these disturbances in Zn and Se homeostasis in long-term dialysis patients remain to be determined.

Zn is an anti-oxidant, has anti-inflammatory properties, and regulates innate and adaptive immune responses, which makes it crucial for resistance to infection [2]. Zn deficient status is associated with immune system disturbances, poor nutritional status, atherosclerosis, high rates of hospitalization due to infections [7,8,9,10], and significant reductions in blood Se concentrations in chronic dialysis patients [11,12]. Conversely, improved Zn status is associated with alleviating oxidative stress, inflammation, dyslipidemia, and malnutrition in dialysis patients [7,13,14,15].

Se has antioxidant properties and protects biological systems from oxidative damage. Se also attenuates inflammatory responses and is required for immune system function [2]. Se deficiency contributes to immune system dysfunction, increases the death from infections, and is a higher cardiovascular disease risk for dialysis patients [16,17]. Se supplementation can decrease oxidative stress and inflammatory status, thus improving the nutritional status of dialysis patients [18]. Thus, Zn and Se appear to have positive synergistic effects [19] and maintaining these trace metals’ homeostasis is critical for dialysis patients.

Patients undergoing long-term dialysis are also at risk for excess concentrations of non-essential trace metals [11,12]. In particular, aluminum (Al) is non-essential for life processes and may act as a toxicant; higher Al blood concentrations have been found in dialysis patients compared to healthy subjects [20,21]. Higher Al concentrations, and lower Zn and Se status, and increased oxidative stress and inflammation have been observed [21]. Oxidative phenomena can be triggered by Al, which decreases both Zn and Se status and promotes inflammatory processes in animals [22,23]. In rat models, administering Se or Zn have protected tissues from Al-induced inflammation and oxidative damage [24,25]. Thus, elevated Al concentrations may be related to interactions with Zn and Se and result in a further increase in oxidative stress and inflammation in patients.

Few studies have examined whether reducing oxidative stress will affect concentrations of Al in the circulation. Improving blood Zn status reduces oxidative stress and attenuates the progression of kidney damage, which possibly contributes to ameliorating plasma Se depletion and Al retention in ESRD patients on long-term dialysis. In this study, we examined the effects of Zn supplementation on plasma Al and Se status and oxidative stress in both plasma and peripheral blood mononuclear cells (PBMCs) in Zn-deficient, dialysis patients with higher than normal Al concentrations.

## 2. Experimental Section

### 2.1. Patients

Between January and December 2007, Zn-deficient patients (plasma Zn concentrations less than 80 mg/dL) with higher than normal plasma Al status (more than 30 μg Al/L) [21] on regular CAPD or HD from the renal dialysis unit were enrolled. All patients with less than 6 months of follow-up, had a recent episode of peritonitis within one month, or patients who were more than 70 years of age were excluded. CAPD patients used 2.5% glucose bags for 3 exchanges and a 7.5% glucose bag for 1 exchange daily. HD patients regularly received a minimum of 12 h HD treatment per week (3 sessions of 4 h each) with a high-flux membrane dialyzer. In all participants, none had smoking, alcohol consumption, and gastrointestinal disorders, liver diseases, cancers, mental retardation or dementia, psychiatric illness, received immune suppressant drugs and Al hydroxide phosphate binders, and supplementation with natural herbs, antioxidants, vitamins/minerals, and fish oils. All patients were clinically stable condition as outpatients. In clinical characteristics of patients, hypertension, ischemic heart disease, dyslipidemia, diabetes mellitus, and some drugs use were also recorded.

Patients were divided into two groups according to plasma Al concentrations (HA group; Al > 50 μg/L and Al of > 30 μg/L to ≤ 50 μg/L were classified as MA group). The cut-off levels for Al were selected on the basis of our previous study and the median laboratory value of Al was 50 μg/L [21]. All participants received oral supplementation with 11 mg elemental Zn per day (78-mg Zn gluconate) for two months. Zn was administered after dinner and the daily dose was based on the recommendations as described by Wang *et al*. [26]. In addition, healthy subjects (control) of similar age and gender who did not receive Zn supplementation were included. All subjects signed an informed consent statement prior to inclusion in the study. The study protocol was approved by the ethics in human research committee of Kuang Tien General Hospital.

### 2.2. Biochemical Analyses

Peripheral blood samples were drawn in the morning from participants between 07:30 and 09:00, after an overnight fast of 12 h at baseline, at one month, and again after two months of Zn treatment. Plasma concentrations of albumin, hemoglobin, blood urea nitrogen (BUN), creatinine, triglyceride, and glucose were determined using a Hitachi 7050 automatic analyzer (Hitachi Corp., Tokyo, Japan). Urea clearance calculated as Kt/V, where K is a constant, t is time, and V is total body water. The estimated glomerular filtration rate (GFR) was obtained using the Modification of Diet in Renal Disease (MDRD) study equation [27].

### 2.3. Determination of Al, Zn, and Se

Plasma Al concentration was determined by atomic absorption spectrophotometer (932AA, GBC, Australia) with a graphite furnace. The instrument was adjusted to a wavelength of 309.3 nm, with a slit of 0.5 nm, and used a hollow cathode Al lamp. The lamp current was 8.0 mA, the integration time was 1 s, and double-beam and D2-background correction were used. The concentrations of plasma Zn were also measured by flame atomic absorption spectrophotometer (932 plus, GBC, Australia) using an air-acetylene flame without background correction at 213.9 nm [28]. Triplicate absorbance readings were taken for each sample in the peak-height mode. Samples were digested in a H_2_O_2_/HNO_3_ mixture in a start D microwave-assisted digestion system (Milestone Microwave Labstation ETHOSD) and subsequently brought up the volume with double deionized water.

For the determination of plasma Se, atomic absorption spectrophotometry with the accessory hydride generation system (HG 3000, GBC, Australia) was used. Samples were digested for a total of 10.5 h with initial temperature of 60 °C for 1–1.5 h, followed by increasing temperatures on 20 °C increments and finally heated up to 225 °C for 2 h in a mixture of 3.2 mL nitric acid (16 N), and 0.8 mL concentrated perchloric acid to convert all Se species to selenate. The reduction of selenate was completed within 30 min at a block temperature of 120 °C [29]. Accuracy of this method was confirmed by comparing to serum reference materials (level 2, NO0371, Seronorm, Nycomed, Oslo, Norway).

### 2.4. Measurement of Plasma Oxidative Stress

The extent of lipid peroxidation was determined by assaying the formation of malondialdehyde (MDA). Briefly, plasma samples were mixed with 3% sodium dodecyl sulfate, 0.1 N HCl, 10% phosphotungstic acid, and thiobarbituric acid, and then incubated at 95 °C for 60 min. The *n*-butanol was added and the mixture was shaken vigorously. After centrifugation at 12,000× *g* and 4 °C for 15 min, the absorbance of the upper layer was read at 530 nm with excitation at 485 nm [4]. The MDA levels were calculated using the 1,1,3,3-tetraethoxypropane as standards.

### 2.5. ROS Concentrations in Isolated PBMCs

PBMCs of all participants were isolated from peripheral blood by FICOLL density gradient centrifugation and re-suspended with RPMI 1640 medium. PBMCs (5 × 10^5^) were incubated with 20 ng/mL of dichlorodihydrofluorescein diacetate (H2DCF-DA) for one hour. The whole-cell ROS concentrations were quantified, and the results were expressed as ROS concentrations per total protein concentration (pm DCF/mg protein). Protein content was determined using the Coomassie protein assay (Pierce, Rockford, IL, USA) with bovine serum albumin as the standard.

### 2.6. Measurement of Plasma Antioxidant Enzyme Activities

The plasma superoxide dismutase (SOD) activity was determined with SOD assay kits (Cayman, Ann Arbor, Michigan, USA); one unit was defined as the amount of enzyme necessary to produce 50% dismutation of the superoxide radicals. The absorbance was measured at 450 nm. The activity of SOD was expressed in unit per mL. In addition, GPx activity was determined using a commercial kit supplied by Cayman Chemical (cat #703102). Oxidized glutathione (GSSG) produced from reducing reactive oxygen species was recycled by NADPH and glutathione reductase to reduced glutathione (GSH). Therefore, the rate of NADPH consumption was utilized as a measurement of the rate of GSSG formation. The plasma samples were mixed with the stock solution containing NADPH, GSH and excess glutathione reductase, and incubated at 37 °C for 5 min, followed by addition of 20 μL of cumene hydroperoxide as a substrate. The GPx activities were expressed as nmol NADPH oxidized/min/mL.

### 2.7. Statistical Analysis

Each value was expressed as the mean ± SD or medians (inter-quartile range, IQR), depending on the normality of data distribution (Shapiro-Wilk test). Comparisons of different variables were made by chi-square test, student’s *t*-test, one-way repeated measures analysis of variance (ANOVA), or Friedman repeated measures ANOVA on ranks, as appropriate. A two-tailed *p* value < 0.05 was considered statistically significant. In addition, Pearson’s or Spearman’s correlation coefficients were performed to identify correlations of blood variables.

## 3. Results

### 3.1. Clinical Results

For clinical characteristics of patients, diabetes mellitus (HA 41%; MA 40%), hypertension (HA 20%; MA 25%), ischemic heart disease (HA 12%; MA 15%), and dyslipidemia (HA 25%; MA 30%) were recorded. In addition, the percentages of some drugs used, such as insulin (HA 17%; MA 20%), sulfonylurea (HA 25%; MA 20%), Ca^2+^ channel antagonists (HA 26%; MA 25%), and beta-blocker (HA 12%; MA 15%), were similar between the two groups of patients.

Relative to healthy controls, Zn-deficient patients had high BUN and plasma creatinine concentrations (*p* < 0.05), but had low hemoglobin and albumin concentrations (Table 1). Compared with MA group patients, patients in HA group had lower baseline hemoglobin concentrations. The concentrations of all other variables tested were comparable between the two patient groups. There were no particular subjective symptoms observed for the Zn-treated patients.

**Table 1:** Baseline characteristics of the study group subjects ^1,2^^,3^.

	HA Group Patients ( *n* = 24)	MA Group Patients ( *n* = 20)	Healthy Subjects ( *n* = 25)
*Basic clinical characteristics*			
Age (years)	53 ± 6	55 ± 7	53 ± 7
Gender (M/F)	9/15	8/12	9/16
Dialysis modality (CAPD/HD)	19/5	15/5	-
Dialysis duration (years)	5 ± 2	6 ± 1	-
Body mass index, BMI (kg/m^2^)	22 ± 3	22 ± 2	24 ±3
BUN (mg/dL)	52 (41–74) ^b^	56 (40–78) ^b^	20 (12–26) ^a^
Creatinine (mg/dL)	11 (9–13) ^b^	11 (10–12) ^b^	2 (2–3) ^a^
Albumin (mg/dL)	3.2 ± 0.3 ^a^	3.3 ± 0.4 ^a^	4.8 ± 0.5 ^b^
Hemoglobin (gm%)	9.2 ± 1.1 ^a^	10.0 ± 1.0 ^b^	16.1 ± 2.1 ^c^
nPNA (g/kg/day)	0.9 ± 0.1	1.0 ± 0.3	-
GFR (mL/min/1.73 m^2^)	3.6 (3.2–4.4)	4.0 (3.6–5.3)	-
Kt/V (/week)	2.0 ± 0.4	2.1 ± 0.5	-
*Essential and non-essential trace metals*			
Zn (mg/dL)	49.2 ± 7.7 ^a^	54.2 ± 9.8^ a^	98.9 ± 6.4 ^b^
Al (μg/L)	73.3 (57.2–86.3) ^c^	41.7 (35.5–46.3) ^b^	25.9 (18.5–27.8)^ a^
Se (ng/mL)	46.1 ± 10.2 ^a^	53.2 ± 16.9 ^a^	106.4 ± 12.7 ^b^
*Oxidant-antioxidant status*			
MDA (nmol/L)	6.9 (6.4–7.7) ^c^	6.0 (5.4–6.8) ^b^	2.9 (2.5–3.4) ^a^
ROS in PBMCs (pm DCF/mg prteoin)	315.2 ± 58.1 ^c^	272.5 ± 77.9 ^b^	105.3 ± 45.3 ^a^
GPx (nmol/min/mL)	48.8 ± 6.3 ^a^	50.5 ± 8.4 ^a^	85.2 ± 6.1 ^b^
SOD (U/mL)	3.1 (3.0–3.4) ^a^	3.4 (3.0–4.7) ^a^	8.3 (7.4–9.1) ^b^

^1^ Values are mean ± SD or medians (IQR). ^2^ Values in the same row with different superscripts (a,b,c) are significantly different (*p* < 0.05). ^3^ HA group = baseline plasma Al > 50 μg/L; MA group = baseline plasma Al > 30 to ≤ 50 μg/L. BUN = blood urea nitrogen; nPNA = protein equivalent of total nitrogen appearance normalized to body weight; GFR = glomerular filtration rate; Kt/V = clearance of urea normalized to total body water. Se = selenium; Zn = zinc; Al = aluminum; MDA = malondialdehyde; ROS = reactive oxygen species; SOD = superoxide dismutase; GPx = glutathione peroxidase.

**Figure 1:**
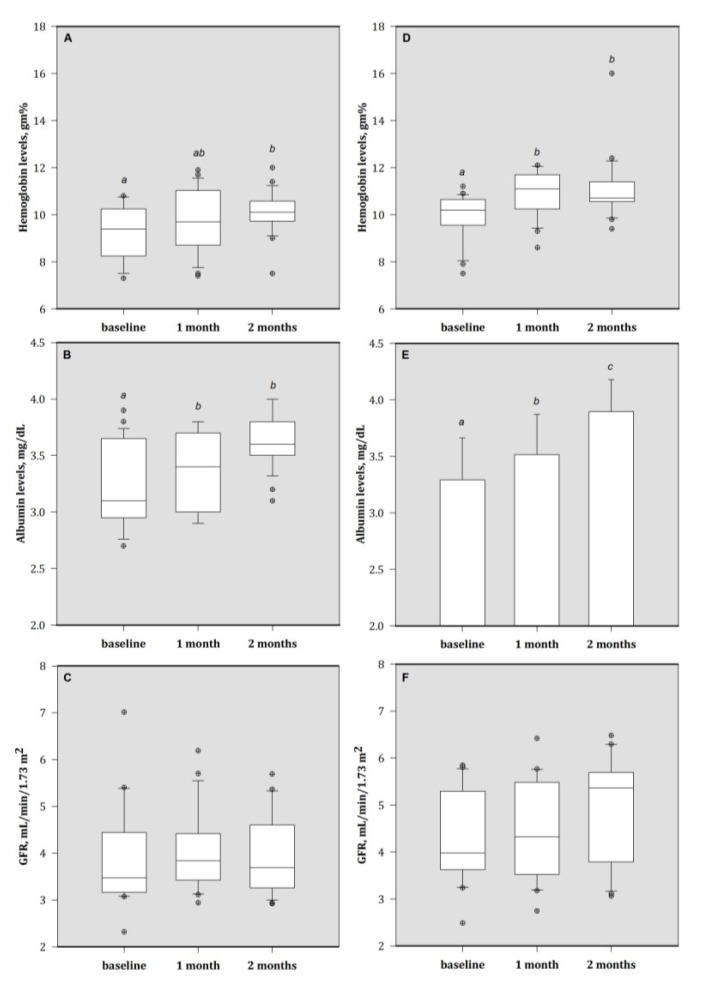
Plasma concentrations of (**A**) hemoglobin, (**B**) albumin, and (**C**) GFR in HA group patients; (**D**) hemoglobin, (**E**) albumin, and (**F**) GFR in MA group patients. Bars are mean (SD) or median (IQR), depending on the normality of data distribution;bar with different superscripts are significantly different (*p* < 0.05). Values above the box plots are outliers. HA group = baseline plasma Al > 50 μg/L; MA group = baseline plasma Al > 30 to ≤ 50 μg/L. 1 month = 1 month of treatment; 2 month = 2 months of treatment (end).

After two months, patients who received Zn supplementation had higher hemoglobin and albumin concentrations compared to their baseline values (Figure 1). As compared with baseline values, patients in the MA group had non-significantly higher values of GFR after two-month Zn treatment.

### 3.2. Plasma Metal Status

**Figure 2:**
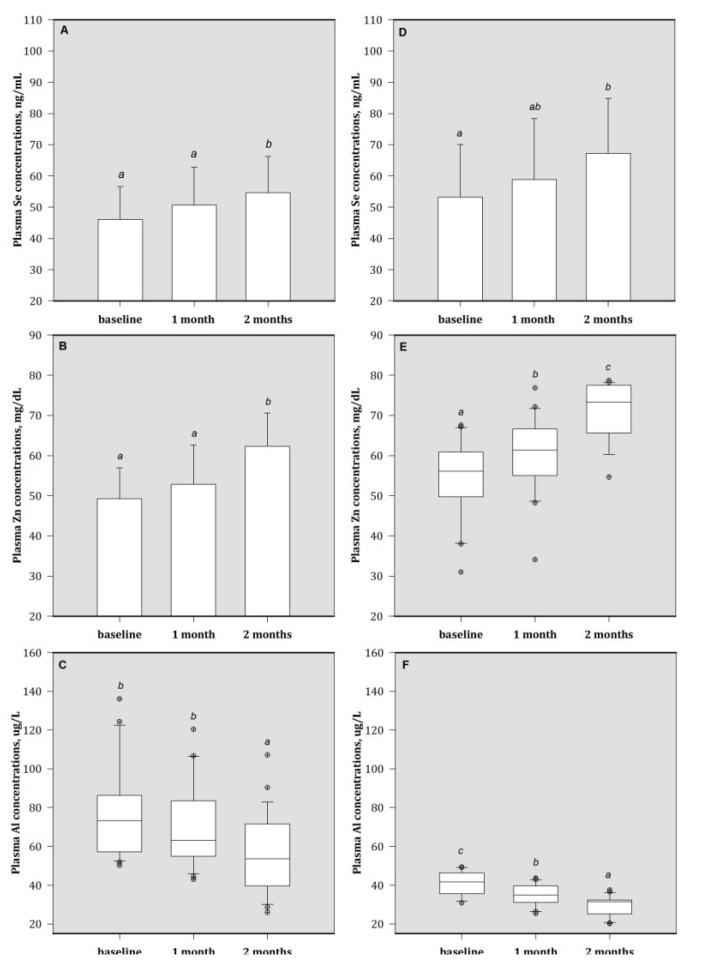
Plasma concentrations of (**A**) Se, (**B**) Zn, and (**C**)Al in HA group patients; (**D**) Se, (**E**) Zn, and (**F**) Al in MA group patients. Bars are mean (SD) or median (IQR), depending on the normality of data distribution; bar with different superscripts are significantly different (*p* < 0.05). Values above the box plots are outliers. HA group = baseline plasma Al > 50 μg/L; MA group = baseline plasma Al > 30 to ≤ 50 μg/L. Se = selenium; Zn = zinc; and Al = aluminum.1 month = 1 month of treatment; 2 month = 2 months of treatment (end).

At baseline, patients had low plasma Se concentrations than the healthy subjects (Table 1). At the end of study, these patients in MA group had higher Se concentrations compared to their baseline values (Figure 2). Patients in both MA and HA groups showed significantly increases in plasma Zn concentrations after the two-month treatment. The elevation in plasma Zn concentrations that was faster for MA group comparative to the HA group. At the end of Zn treatment, patients had decreased plasma Al concentrations compared to their baseline values.

### 3.3. Oxidative Stress Status

Baseline concentrations of the plasma oxidative product MDA were higher in patients compared to healthy subjects (Table 1). Further, plasma MDA concentrations were highest in the HA group patients.

**Figure 3:**
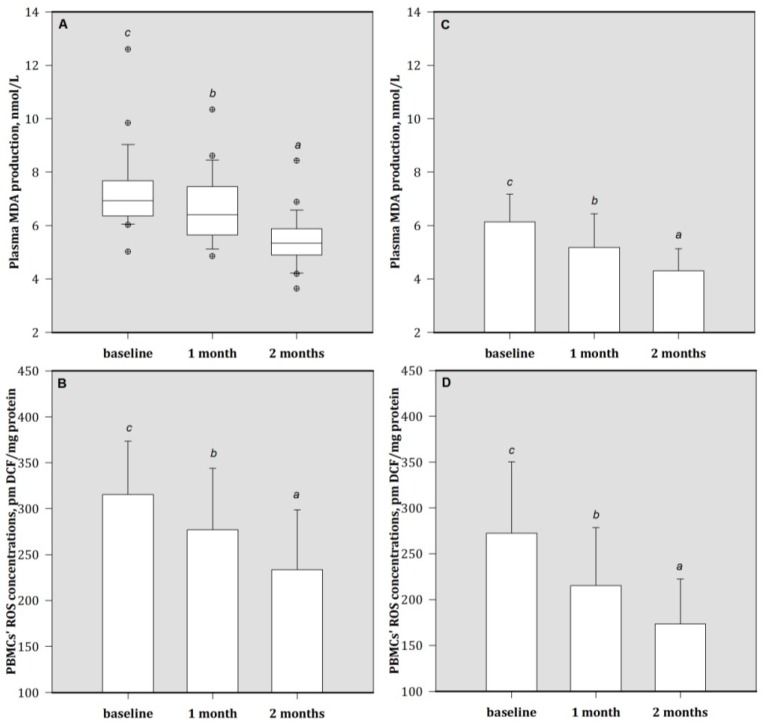
The concentrations of (**A**) plasma MDA and (**B**) peripheral blood mononuclear cells (PBMCs)’ ROS in HA group patients; (**C**) plasma MDA and (**D**) ROS concentrations in MA group patients. Bars are mean (SD) or median (IQR), depending on the normality of data distribution; bar with different superscripts are significantly different (*p* < 0.05). Values above the box plots are outliers. HA group = baseline plasma Al > 50 μg/L; MA group = baseline plasma Al > 30 to ≤ 50 μg/L. MDA = malondialdehyde; ROS = reactive oxygen species.1 month = 1 month of treatment; 2 month = 2 months of treatment (end).

For Zn-supplemented patients, decreases in plasma MDA concentrations were observed after one month. At the end of Zn treatment, these patients had significantly decreased MDA concentrations compared to their baseline values (Figure 3). Additionally, similar trends were found for the ROS concentrations in PBMCs of both patient groups. Compared to HA group, patients in MA group had lower ROS concentrations, at the end of the study (data not shown).

**Figure 4:**
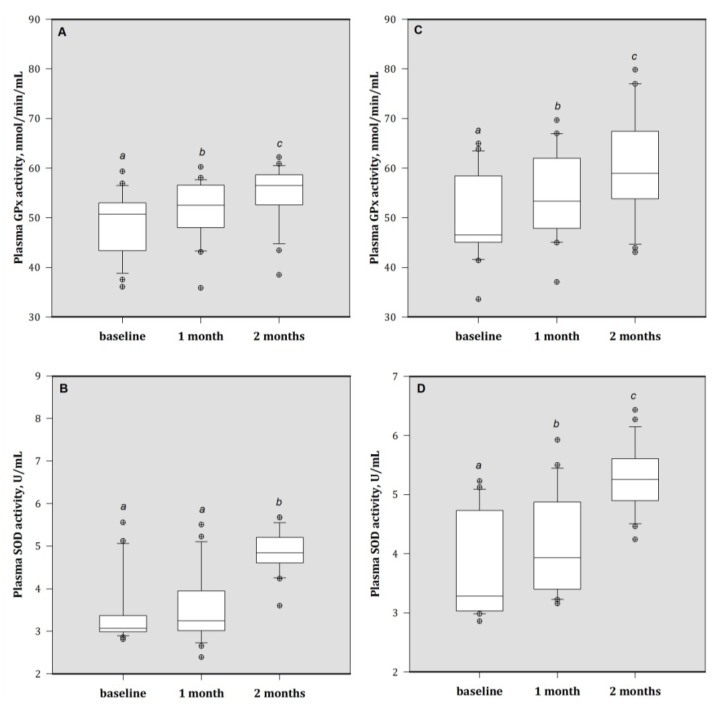
The plasma activities of (**A**) GPx and (**B**) SOD in HA group; the plasma (**C**) GPx and (**D**) SOD activities in MA group. Bars are median (IQR), and with different superscripts are significantly different (*p* < 0.05). Values above the box plots are outliers. HA group = baseline plasma Al > 50 μg/L; MA group = baseline plasma Al > 30 to ≤ 50 μg/L. GPx = glutathione peroxidase; SOD = superoxide dismutase.1 month = 1 month of treatment; 2 month = 2 months of treatment (end).

At baseline, there were significant reductions in antioxidant enzyme GPx and SOD activities in these patients. Zn supplementation for the HA group patients did not change the activity of SOD after one month. After two months, these patients in both MA and HA groups showed increased both GPx and SOD activities at the end of study (Figure 4).

### 3.4. Correlations between Al, Other Metals, and Oxidative Stress

After two months of Zn administration, plasma Zn concentrations were correlated with Al, Se, MDA concentrations, and SOD activities in both HA and MA groups (Table 2). Further, partial correlation analysis indicated that the correlation between the plasma Zn and plasma Se was non-significant when the effect of Al was eliminated.

**Table 2:** Correlations between plasma Zn with Al, Se, MDA concentrations, and SOD activities in patients after two months of Zn supplementation ^1,2,3^^,4^.

	HA Group Patients			MA Group Patients	
Zn-MDA	*r* = −0.49			*r* = −0.48	
Zn-SOD	*r* = 0.51	*p* < 0.05		*r* = 0.58	*p* < 0.05
Zn-Al	*r* = −0.43			*r* = −0.50	
Zn-Se	*r* = 0.40			*r* = 0.52	
	*r*_12.3_ = −0.46	*p* < 0.05		*r*_12.3_ = −0.45	*p* < 0.05
	*r*_13.2_ = 0.29	*p* = 0.25		*r*_13.2_ = 0.41	*p* = 0.05

^1^ HA group = baseline plasma Al > 50 μg/L; MA group = baseline plasma Al > 30 to ≤ 50 μg/L. ^2^ Zn-MDA = correlation between Zn and MDA; Zn-SOD = correlation between Zn and SOD. ^3^ Zn = x_1_; Al = x_2_; Se = x_3_; * r*_12.3_ = partial correlation between x_1_ and x_2_ eliminating the linear effect of x_3_.^4^ Al= aluminum; Se=selenium; Zn=zinc; MDA = malondialdehyde; SOD = superoxide dismutase.

## 4. Discussion

Recent studies have shown that Zn supplementation increases serum Zn concentrations, improves taste acuity and the protein catabolic rate [14,30], and raises cholesterol, low-density lipoprotein and high-density lipoprotein concentrations [31,32,33] in patients with chronic dialysis. Moreover, plasma concentrations of MDA and pro-inflammatory cytokines have been reported to be lower in dialysis patients treated with Zn supplements [7,34].

In this preliminary investigation, Zn-deficient dialysis patients with higher plasma Al concentrations had low plasma Se concentrations along with decreased activities of antioxidant enzyme GPx than the healthy controls. Several studies have shown that circulating Se concentrations in patients undergoing hemodialysis and CAPD were lower than those in healthy controls [16,17,35], although conflicting results have been reported [36]. Low Se status may be attributed to reduced Se intake, malnutrition, impaired absorption due to high levels of pro-inflammatory cytokine, diminished Se retention due to chronic oxidative stress, or increased loss of Se in the dialysis effluent [2,37]. Further, GPx is a Se dependent protein and circulating GPx activities are responsive to Se status in the biological systems [38]. Kidneys can accumulate the highest amounts of Se and are the major source of plasma GPx [39]; thus, the levels of both Se and GPx can be reduced in ESRD patients.

A negative interaction between plasma Al and Se in hemodialysis patients has been found [21]. In the present study, plasma Al status appears to have a potential effect on the correlation between Zn and Se. Thus, increased Al appeared to suppress Se, which may have been another contributing factor to lower plasma Se status in these dialysis patients. Although Al intoxication can be avoided by water purification, these patients undergoing long-term dialysis still have significant increases in circulating Al concentrations [20,21,40]. In a rat model, Al administration also disrupted the distribution of Se and decreased GPx activity, which increased oxidative damage in target tissues [23]. Al exposure increases oxidative stress and pro-inflammatory cytokine concentrations that are ameliorated by Se treatment [24], whereas Se does not have a protective effect against the toxic effects of Al [41].

Roles for Al have been proposed in dialysis encephalopathy, microcytic anemia, arterial stiffness, and osteomalacia in ESRD patients. Possible sources of Al overload for dialysis patients include Al hydroxide phosphate binders, diets that are high in either cooked or processed foods, contamination of dialysis machines, and reduced urinary excretion of Al in long-term dialysis patients [21,42]. Furthermore, another major contributor to accumulation of Al was uremic compounds in patients undergoing chronic dialysis [43]. Kinetic analysis from peritoneal patients has shown that the half-life of Al elimination is probably approximately seven years [44]. The chelating agent deferoxamine can be used to remove excess Al, whereas some deleterious effects were noted [45]. Apparently, effective Al reduction in the circulation is likely to be an important therapeutic target.

Compared to healthy subjects, patients also showed increased plasma production of MDA, higher intracellular ROS concentrations in PBMCs, and lower antioxidant enzyme SOD activities. Some studies indicated that chronic dialysis patients had significantly higher oxidative stress [3,4,46]. Zn plays a critical structural role for SOD and can stabilize biological membranes to decrease their susceptibility to oxidative stress. A pronounced decrease in plasma Zn concentrations among patients with increased Al concentrations was noted in patients with chronic renal insufficiency [47]. Al hydroxide phosphate binders used by patients can interfere with Zn status [48]. In a rat model, the interaction between Al and Zn interfered with SOD activities [49]. Thus, higher plasma Al concentrations may interfere with both Zn and Se homeostasis, which consequently leads to low plasma Zn and Se concentrations and high oxidative stress in long-term dialysis patients.

In our present preliminary results, Zn administration decreased plasma Al concentrations, which was linked to lower MDA concentrations and higher SOD activity. Once plasma Zn status had increased, this may have contributed to ameliorating their worsening renal function by reducing Al retention in these patients. Increased Zn status improves the intestinal barrier integrity [50] may decrease Al concentrations in the circulation.

On the other hand, hemoglobin and albumin status are influenced by numerous factors but are widely used as markers of nutritional status, and as strong predictors of mortality and morbidity [3]. Our present results show that patients receiving Zn had higher plasma concentrations of hemoglobin and albumin. Zn supplementation may improve the acuity of taste and smell and gastrointestinal function, increase food intake, and reduce protein-energy wasting [2,30,51]. Thus, The Zn-deficient patients who receive Zn supplementation have improvements in their oxidant-antioxidant balance and nutritional status [13,14], which likely contributes to their increased plasma Se status.

## 5. Conclusions

Long-term dialysis patients with low plasma Zn concentrations had reduced plasma concentrations of Se. Dietary intake data for Se and Zn was not available, which we consider to be a limitation. The study was also limited in comparing the variables based on dialysis modalities and the potential interaction between Al and renal function. The present pilot study is not a randomized trial, the results show that Zn administration increased the initially low plasma Zn concentrations concomitant with Se concentrations in patients and contributed to reducing their high plasma Al concentrations and oxidative stress. Additional Zn supplement may be necessary to alleviate symptoms of long-term dialysis. Further large-scale studies will be needed to clarify the effects of Zn supplement on Al metabolism in long-term dialysis patients.
